# A feeding protocol for delivery of agents to assess development in Varroa mites

**DOI:** 10.1371/journal.pone.0176097

**Published:** 2017-04-27

**Authors:** Ana R. Cabrera, Paul D. Shirk, Peter E. A. Teal

**Affiliations:** 1 University of Florida, Entomology and Nematology Department, Gainesville, Florida, United States of America; 2 USDA-ARS Center for Medical, Agricultural and Veterinary Entomology, Gainesville, Florida, United States of America; CNRS, FRANCE

## Abstract

A novel feeding protocol for delivery of bio-active agents to Varroa mites was developed by providing mites with honey bee larva hemolymph supplemented with cultured insect cells and selected materials delivered on a fibrous cotton substrate. Mites were starved, fed on treated hemolymph to deliver selected agents and then returned to bee larvae. Transcript levels of two reference genes, actin and glyceraldehyde 3-phosphate dehydrogenase (GAPDH), as well as for nine selected genes involved in reproductive processes showed that the starvation and feeding protocol periods did not pose a high level of stress to the mites as transcript levels remained comparable between phoretic mites and those completing the protocol. The feeding protocol was used to deliver molecules such as hormone analogs or plasmids. Mites fed with Tebufenozide, an ecdysone analog, had higher transcript levels of *shade* than untreated or solvent treated mites. In order to extend this feeding protocol, cultured insect cells were incorporated to a final ratio of 1 part cells and 2 parts hemolymph. Although supplementation with *Bombyx mori* Bm5 cells increased the amount of hemolymph consumed per mite, there was a significant decrease in the percentage of mites that fed and survived. On the other hand, *Drosophila melanogaster* S2 cells reduced significantly the percentage of mites that fed and survived as well as the amount of hemolymph consumed. The feeding protocol provides a dynamic platform with which to challenge the Varroa mite to establish efficacy of control agents for this devastating honey bee pest.

## Introduction

The Varroa mite, *Varroa destructor* (Anderson & Trueman) (Mesostigmata: Varroidae), has become the most serious health problem for honey bees in the United States and worldwide [1–4]. Varroosis along with environmental stressors such as pathogens, poor nutrition and pesticides [5–7] have contributed to high colony losses during the past nine winter seasons due to the parasitism and virus transmission [8–10]. These annual hive losses constitute a significant economic burden to commercial beekeepers who must meet pollination service needs, which contribute an additional estimated at $20.1 billion (€18.6 billion) to the US economy [3].

The Varroa mite is an obligate ectoparasite feeding on the hemolymph of all stages [1, 11] and undergoes a seasonal population increase soon after the peak brood production in a bee colony [12, 13]. The feeding of the Varroa mites can result in lower weights of emerging adult bees from parasitized larval and pupal bees and shorter lifespans of adult bees [1, 11, 14–17]. Varroa mite feeding also leads to a weakening of the bee immune system [18] may facilitate the transmission of multiple bee viruses. However, it appears the mutualistic symbiosis between Varroa mites and Deformed Wing Virus (DWV) can lead to an induced immunosuppression in honey bees that increases mite feeding and reproduction [19] which fuels the transmission of this highly virulent strain of the DWV [20, 21]. Additionally, the dissemination and spread of parasites/diseases contributing to this global epidemic has been facilitated by the worldwide commercial trade and transport of the honey bees [22–24]. Thus, the aggregate load from Varroa mite feeding, reduced immunity, increased viral transmission as well as lethal and sublethal intoxication effects of pesticides that are placed on a colony contributes to winter colony losses that are being experienced in North America and Europe [2, 6, 23–27].

Reproduction of the Varroa mite is triggered upon entry of a phoretic mite into a brood cell containing a pre-molting last instar larva [1, 4]. A phoretic mite is carried on the host worker bee within the hive until a suitable brood cell is detected and then the mite leaves the host and quickly invades the cell: 40–50 h before capping in drone cells and 15–20 h in worker cells [28–30]. Initiation of oogenesis is observed within 6 h after cell capping [31, 32]. Vitellogenesis is initiated within 10 h after cell capping as the vitellogenin transcripts become elevated [33], the large lipid transfer protein (LLTP) transcripts are depressed [34] and yolk spheres can be observed accumulating within the oocyte [35]. The first egg is laid approximately 70 h after cell capping [32, 36] and is a haploid male [37]. The male egg is followed by as many as 5 female eggs in worker cells and 6 in drone cells [13, 38, 39]. Adult Varroa mites escape the cell along with the newly eclosed adult bee and seeks out and infest nurse bees for initial transport [40–42].

Reportedly, the Varroa mite can survive away from the bee from between 18 to 70 h depending on the substrate [43] and possibly up to 6 days if the mite resides on flowers [44, 45]. However, their high rate of metabolism leads to a 8.1% utilization of their wet weight per hour for the first six hours of starvation all based on a reduction in nutritional reserves [46]. This nutritional loss was not sustainable as more than 95% of the starving Varroa mites died within 36 h of removal from the host [46].

Administering biological materials to the mites has proved to be problematic due to the difficulties in sustaining the Varroa mites while off of the host thus hindering research progress into development, reproduction and virus transmission [47]. Application of RNA interference (RNAi) has been achieved by soaking the mites in an 0.9% saline solution containing a double stranded RNA (dsRNA) sequence with return to a bee pupae has resulted in knockdown of mu-class glutathione S-transferase, B-type allatostatin, a member of the crustacean hyperglycaemic hormone (CHH), and pheromone receptor transcription factor (PRTF) [48–50]. Alternatively, feeding of the Varroa mites to deliver biologicals has been less successful and has required some form of membrane such as stretched Parafilm [51, 52]. The most successful feeding assay utilized a chitosan membrane that provided a feeding substrate and barrier between the nutrient media and the mites [53]. Although, feeding on various nutritive media was assessed using chitosan membranes, no delivery of biologicals was determined. Here, we describe a novel short-period feeding protocol based on dispensing larval bee hemolymph containing treatment materials to multiple Varroa mites without the use of a membrane substrate. Validation of the feeding protocol conditions by assessing mite survival and changes in transcript levels of genes regulated during reproduction demonstrates that the protocol does not compromise the Varroa mite physiology and can be used to deliver agents such as hormone analogs, plasmids, dsRNA and other chemicals.

## Materials and methods

### Honey bee and Varroa mite cultures

Hives of *Apis mellifera* were maintained at CMAVE, Gainesville, FL, were kept without treatment for Varroa mites or other pest for more than two years to have pesticide-free honey bee populations. Consequently the hives were naturally infested with *Varroa destructor*. Varroa mites were collected as needed from drone or worker cells directly or from adult bees by the sugar shake method using powder sugar [54].

### Collection and preparation of honey bee larva hemolymph

Fifth instar honey bee larvae were removed from frames and placed in groups of 3–4 larvae in a 50 ml conical tube custom fitted with nylon mesh approximately 1.5 cm from the tube opening. After a small puncture was made with a fine forceps in each of the larvae, the hemolymph was collected by centrifugation (2 min, 3 x g at 4°C). The hemolymph was transferred and pooled in a 1.5 ml Eppendorf tube on ice until 1.0 ml total was collected (20–25 larvae). To inhibit oxidation, ascorbic acid (A1300000; Sigma-Aldrich) was added to the hemolymph to a final concentration of 10 mM for drone larvae hemolymph or 15 mM for worker larva hemolymph. A higher concentration was needed to prevent oxidation of the worker larva hemolymph compared to drone larva hemolymph (data not shown). A final concentration of 2 μg/ml ampicillin was also incorporated into the hemolymph to prevent bacterial growth. The hemolymph was used immediately for a feeding protocol unless otherwise indicated. In order to assess stability, additional hemolymph samples were placed at -20°C for 3, 6 or 9 weeks after addition of ascorbic acid.

### Varroa mite feeding protocol

The feeding protocol was conducted in an inverted flat-cap 2 ml microcentrifuge tube (Fisher Scientific) (S1 Fig). The barrel of the tube was puncture five times to provide ventilation and the cap contained a small piece of a sterile cotton ball (2 ± 0.5 mg) (22-456-885; Fisher Scientific). The prepared treatment solutions or hemolymphs were dispensed onto the cotton and then 10 to 15 Varroa mites were added prior to closing the tube. The assay tubes were placed in a modular incubation chamber (Billups-Rothenberg, Inc.) with a moist paper towel to provide humidity and the chamber was placed in an incubator at 28 ± 1°C with 24 h darkness.

### Feeding dyes

To identify a food dye that could be included in the hemolymph preparations and not interfere the feeding by the Varroa mites, various dyes were tested at a 1–2% final concentration and included Royal blue icing color (Wilton Enterprises), pink DayGlo stain (DAYGLO Color Corp.), Indigo (V001073; Sigma-Aldrich), Neutral Red (N4638; Sigma-Aldrich) and Rose Bengal (198250; Sigma-Aldrich). The Royal blue icing color was selected for initial experiments because it is a non-toxic certified food additive. Feeding was confirmed by visual inspection to establish the presence of the dye within the gut of the mite and those mites were assessed as percent fed of the total mites in the experimental group. The mites that survived the feeding period were assessed as percent survived of the total mites in the experimental group regardless whether feeding could be confirmed. The mites that were confirmed to have both fed and survived the feeding period were assessed as percent fed/survived of the total mites in the experimental group.

### Treatment of Varroa mites with Tebufenozide

Tebufenozide, an ecdysone analog, was selected for evaluation since evidence suggests that ecdysteroids regulate reproduction in the Acari [55–58]. A stock solution of Tebufenozide (31652; Sigma-Aldrich) was prepared in dimethyl sulfoxide (DMSO) (AA42780AK; Fisher Scientific) and then diluted with the prepared bee larva hemolymph to obtain the following final concentrations: 0.1, 10 and 1000 ng/μl. Control groups included both an untreated hemolymph and a hemolymph containing DMSO without Tebufenozide. After 24 h of feeding, the Varroa mites that had fed were collected and stored at -80°C until processed. Total RNA isolation, cDNA synthesis and qPCR to assess transcript levels of *Vd*dib, *Vd*LLTP, *Vd*shd, *Vd*spo, *Vd*Vg1 and *Vd*Vg2 were conducted as described below.

### Insect cell lines

*Bombyx mori* Bm5 cells were maintained in Grace’s insect medium (11605–094; Thermo Fisher Scientific) supplemented with 10% fetal calf serum (FCS) (S12450H; Atlanta Biologicals) at 24°C. *Drosophila melanogaster* S2 cells were maintained in Schneider’s insect medium (21720–024; Thermo Fisher Scientific) supplemented with 10% Fetal Calf Serum (SIMC) at 24°C. The Bm5 and S2 cells were grown in 25 cm^2^ polystyrene culture flasks (Corning Inc.) and media were exchanged weekly. Doubling time for the Bm5 cells under these conditions was approximately 72 h and for the S2 cells was approximately 12 h. The cells were grown to confluency, scraped, removed to a 15 ml conical culture tube (Corning Inc.) and pelleted by centrifugation (120 x g, RT). The medium was removed and the cells were resuspended in their respective growth medium at 2.7 x 10^5^ cells per ml. The cell suspension was combined with the prepared larval hemolymph (as above) in a 2:1 (hemolymph: cells) ratio. As an internal standard, a pGem-Vdactin plasmid (1 x 10^8^ copies) was added to the hemolymph-cell suspension before applying to the cotton dispenser.

### Quantitative PCR (qPCR) and quantitative Real-Time PCR (RT-qPCR)

Transcript levels of disembodied (*Vd*dib;), foraging (*Vd*For), large lipid transport protein (*Vd*LLTP; ACU30143), malvolio (*Vd*Mvl), shade (*Vd*shd), spook (*Vd*spo), subolesin (*Vd*sub), vitellogenin 1 (*Vd*Vg1; JQ974976), vitellogenin 2 (*Vd*Vg2; JQ974977) and the normalizing genes actin (*Vd*Act; AB242568) and glyceraldehyde 3-phosphate dehydrogenase (*Vd*GAPDH) were assessed from whole body samples consisting of 10 mites surviving the feeding protocol. These nine genes were selected based on previous work assessing differential transcription during the mite reproductive phase [33, 34, 59, 60]. Total RNA was isolated from these samples using the RNeasy Mini Kit (Qiagen Inc.), following the manufacturer’s recommendation. A NanoDrop 2000 spectrophotometer (Thermo Scientific) was used to estimate the total RNA concentration, and the ratios from absorbance at 260 and 280 nm and A260 and 230 nm wavelengths were monitored to assess sample quality. The cDNA was synthesized using the SuperScript^™^ III First-Strand Synthesis System kit (12574030; Invitrogen) with 200 ng of total RNA.

The RT-qPCR reactions were conducted using the CFX96 Touch^™^ Real-Time PCR Detection System (Bio-Rad). Each reaction contained 1 μl of cDNA (diluted 1:50 with water), 0.6 μl of 10 μM-forward primer, 0.6 μl of 10 μM-reverse primer, 7.8 μl of water and 10 μl of Sso Advanced^™^ SYBR^®^ green Supermix (1725274; Bio-Rad). Table 1 presents a list of the primers used to conduct reactions to assess transcript levels of *Vd*dib, *Vd*For, *Vd*LLTP, *Vd*Mvl, *Vd*shd, *Vd*spo, *Vd*sub, *Vd*Vg1, *Vd*Vg2 and the normalizing genes *Vd*Act and *Vd*GAPDH [59]. The transcript levels from each biological sample were normalized to the geometric mean for actin and GAPDH which was derived from the transcript levels (Cq values) of *Vd*Act and *Vd*GAPDH (geometric mean = √(Cq_actin_) x (Cq_GAPDH_)) [61]. Prior to use, the efficiencies of each primer set were evaluated by standardization against a dilution series (10^2^, 10^4^, 10^6^ and 10^8^ copies /μl) of each respective plasmid pGEM-T Easy (A1360; Promega) containing an insert of the coding region for *Vd*dib, *Vd*For, *Vd*LLTP, *Vd*Mvl, *Vd*shd, *Vd*spo, *Vd*sub, *Vd*Vg1, *Vd*Vg2, *Vd*Act or *Vd*GAPDH. Plasmid DNA (10^2^, 10^4^, 10^6^ and 10^8^ copies /μl) with a fragment of either actin or GAPDH (housekeeping genes) was used to generate a standard curve to assure quality for each RT-qPCR reaction series. Each experimental group of 10 mites surviving the feeding protocol had six biological replicates with three technical replicates. The ΔΔCt value was obtained using the average ΔCt value from *Vd*spo-phoretic mite as the reference group.

**Table 1:** Primer sequences used for real-time PCR of selected Varroa mite genes.

*Primer*	Sequence	Tm (°C)	Primer pair efficiency
*Disembodied*			
*Vd*dib-F	5’-GGAAACGGCAACAGTTCTTC-3’	54.4	98%
*Vd*dib-R	5’-CCTGCCAGGAAAAGATCCAT-3’	55.0	
*Foraging*			
*Vd*For-F	5’-TCGCTGCCCGCGATCAACAAA-3	62.4	102%
*Vd*For-R	5’-GGCGAGAATACCTTGGCGGCAT-3	62.2	
*Large Lipid Transport Protein*			
*Vd*LLTP-F	5’-TGGCACAGCCCCCGTACATT- 3’	64.3	98%
*Vd*LLTP-R	5’-TCGTGTCCGAACGCGCTCAA- 3’	65.1	
*Malvolio*			
*Vd*Mvl-F	5’-TCCTCGGTATCTCCTCTGGA-3’	56.3	97%
*Vd*Mvl-R	5’-GGAACAAGGCGATTGATGTT-3’	53.5	
*Shade*			
*Vd*shd-F	5’-ATAGTCGGGGGCATTTTCTC-3’	54.7	104%
*Vd*shd-R	5’-TCGTAGATTGTCGCTTGCAC-3’	55.3	
*Spook*			
*Vd*spo-F	5’-CGGGTGCTGACCATAAGAAG-3’	55.6	107%
*Vd*spo-R	5’-CGTGGCTACGTGTGGAATTA-3’	54.9	
*Vitellogenin1*			
*Vd*Vg1-F	5’-CATTGTTGCCGCACACACCGT-3’	64.8	98%
*Vd*Vg1-R	5’-AATGGCCAGCGCGTCTACCT-3’	64.4	
*Vitellogenin2*			
*Vd*Vg2-F	5’-TGGCGTCACGGGACTGAACA-3’	64.2	97%
*Vd*Vg2-R	5’-TGCGGTAGCGCGAACAACGA-3’	65.4	
*Subolesin*			
*Vd*sub-F	5’-GGGTCTTCGAGGGAACAATA-3’	54.8	99%
*Vd*sub-R	5’CGGAACTTGCAATCAGTTCA-3’	53.4	
*Actin*			
*Vd*Act-F	5’-TTGCTGACCGTATGCAGAAA-3’	54.6	94%
*Vd*Act-R	5’-CCGATCCAGACGGAATACTT-3’	54.0	
*GAPDH*			
*Vd*GAPDH-F	5’-CGCAAGGCCGGTGCCAAAAA-3’	62.1	97%
*Vd*GAPDH-R	5’-ACGAACATTGGCGCATCGGGT-3’	62.3	

Uptake and persistence of plasmid DNA during feeding was assessed by addition of 1 x 10^10^ copies of pGem-Vdactin [33] /μl to the feeding medium. After completion of the feeding protocol, genomic DNA was isolated from samples consisting of 10 surviving Varroa mites using the QIAamp DNA Mini kit (Qiagen) according to the manufacturer’s protocol. The amount of plasmid in the mites was determined by qPCR using the gDNA extract as template with M13F-40 forward primer 5’- GTT TTC CCA GTC ACG AC-3’ and actin (*Vd*Act-R) reverse primer 5’-CGATCCAGACGGAATACTT-3’ [33]. Each mite feeding protocol had 6 biological replicates, and 3 qPCR reactions were performed for each biological replicate (technical replicates). The amount of feeding medium consumed was estimated by determining the quantity of plasmid recovered from the extracted mites based on a Cq value standard curve established from a dilution series of *Vd*Act plasmid DNA (10^2^, 10^4^, 10^6^, 10^8^ copies /μl). From the Cq value of the extracted material, the amount of plasmid DNA that each mite consumed was estimated by adjusting for sample dilution of the recovered DNA and the number of mites per sample (10 mites).

### Statistical analyses

All statistical analyses were carried out using SAS v. 9.4 (Cary, NC). Comparisons of the various diets were made between mites fed with drone larva hemolymph with Royal blue icing color, 10 mM ascorbic acid and 2 μg/ml ampicillin as the reference treatment. Each feeding assay (10–20 mites per microcentrifuge tube) was considered an experimental unit. ANOVA was conducted with PROC GLM to compare the percentage of Varroa mites that fed and survived each dietary treatment, followed by Dunnett’s test for mean comparison to the reference. Transcript levels (ΔCt values) from actin, GAPDH, LLTP, Vg1, Vg2, For, Mvl, Spo, Dib, Shd, and Sub were compared between phoretic mites and mites that were placed on the feeding assay and then kept with bee larvae, using PROC TTEST. To evaluate transcript levels of Vg1, Vg2, LLTP, and the Halloween genes in mites exposed to Tebufenozide, Type I error rate was set at α = 0.05.

## Results

### Establishment of a Varroa mite feeding protocol

Preliminary assessments for delivering a feeding medium were conducted utilizing a clarified homogenate of adult honey bees via different materials. Royal blue icing color was added to the hemolymph at a 2% final concentration to identify mites that fed and survived after 24 h. When compared with all other delivery methods assessed here, the Varroa mites had the highest survival frequency after 24 h when the feeding medium was dispensed on a small piece of a cotton fiber ball (65%) and not through various membranes (S1 Table). Subsequently, all feeding protocols designed to improve the survival frequency of the mites were based on dispersing the feeding medium on a cotton fiber ball.

Unfortunately, the homogenized adult bee medium became heavily oxidized after a few hours and was impractical to use and a more stable and efficient feeding medium was sought. Water alone was used as a baseline control and resulted in a 4.0 ± 9.7% (mean ± SD) feeding and 47.0 ± 24.1% survival after 24 h (n = 10, Table 2). Schneider’s insect medium (SIMC) resulted in a high level of survival (71.7 ± 13%) but there was only a 12.1±10.0% confirmed feeding frequency (n = 8). Overall there were 12.0 ± 11.4% with SIMC that both fed and survived (Fed/Survived). A medium consisting of a bee larva homogenate resulted in a low 4% of the mites that had fed/survived. Similar to homogenized bees, untreated hemolymph melanized after 2–3 hours with only 7.1% of the mites that had fed/survived after 24 h (n = 28); additionally, collection of sufficient amounts of hemolymph from adult nurse bees was impractical to conduct the bioassays.

**Table 2:** Comparison of phoretic Varroa mite survival and feeding on media based on drone larva hemolymph or worker larva hemolymph with addition of ascorbic acid.

Feeding Medium Preparation	n	Fed (%)	Survived (%)	Fed/Survived (%)	Compared With Drone Larva Hemolymph (p-value)
Water	10	4.0 (± 9.7)	47.0 (± 24.1)	3.0 (±9.5)	<0.0001
SIMC	8	12.1 (± 10.0)	71.7 (± 13.9)	12.0 (± 11.4)	<0.0001
Drone larva hemolymph (5 mM Ascorbic acid)	6	36.7 (± 21.6)	46.7(± 22.5)	18.3(± 14.7)	<0.0001
Drone larva hemolymph (10 mM Ascorbic acid)	7	65.7 (± 19.0)	71.4 (± 13.5)	60.0 (± 16.3)	
Drone larva hemolymph (50 mM Ascorbic acid)	6	45.0 (± 17.6)	36.7 (± 20.7)	16.7 (± 8.2)	<0.0001
Worker larva hemolymph (10 mM Ascorbic acid)	8	42.6 (± 14.8)	79.6 (± 7.6)	37.9 (± 12.3)	0.0024
Worker larva hemolymph (15 mM Ascorbic acid)	6	66.1 (± 16.2)	60.0 (± 12.6)	52.9 (± 10.3)	0.4210

Values represent the mean (± SD). ANOVA results indicated there were statistical differences among treatments (F = 24.62, p<0.0001), followed by mean comparison with Dunnett’s test with each treatment and the drone larva hemolymph with 10mM ascorbic acid. Collective information for all data sets are included in S2 Table.

As an alternative source of honey bee material, hemolymph from 5^th^ instar drone larvae was collected by centrifugation and dispensed on the cotton balls. To prevent melanization of the hemolymph, ascorbic acid was added to the drone larva hemolymph at 5, 10 or 50 mM final concentration. The 10 mM ascorbic acid supported the highest percentage of mites that survived 71.4 ± 13.5%, confirmed to have fed 65.7± 19.0% and both fed and survived 60 ± 16.3% for 24 h (n = 7,Table 2). The drone larva hemolymph with 5 mM ascorbic acid treatment melanized after 2–3 h, while the 50 mM treatment had greatly reduced survival rates. In addition to the ascorbic acid, drone larva hemolymph was treated with 2 μg/ml ampicillin which resulted in 60% fed and survived frequency. On the other hand, if ampicillin was not added the percentage of mites that fed/survived was significantly reduced, 25.8%. In order to extend the resource of treated drone larva hemolymph, it was mixed 2:1 with SIMC.

Each drone larva provided approximately 50 μl of hemolymph, but drones were seasonally limiting resource. Consequently, worker larva hemolymph was collected, treated 10 mM ascorbic acid and 2 μg/ml ampicillin and then applied to cotton balls in the feeding protocol. This worker larva hemolymph based feeding medium resulted in a significantly lower percentage of mites feeding and surviving after 24 h (Table 2) which was most likely due to excessive melanization after 3 h. However, when 15 mM ascorbic acid final concentration was added to the worker larval hemolymph, melanization was inhibited and the percentage of mites that fed/survived after 24 h was not significantly different from the drone larval hemolymph (Table 3).

**Table 3:** The comparison of various dyes included in the feeding medium to visually identify mites that had ingested media.

Marker Dye	n	Fed (%)	Survived (%)	Fed/Survived (%)
Royal blue	6	66 (±16.2)	59.1 (±11.9)	52.9 (±10.3)
DayGlow (pink)	6	0	68.6 (±17.2)	0*
Indigo	6	38.3 (±21.4)	56.7 (±26.6)	31.9 (±17.7)*
Neutral Red	7	24.3 (±16.4)	7.1 (±8.2)	1.4 (±3.8)*
Rose Bengal	6	60 (±17.9)	63.3 (±12.1)	38.3 (±7.5)*

The ANOVA indicated statistical differences among treatments (F = 33.20, p<0.0001), and the Dunnett’s test was used for mean comparison among dyes compared to the reference dye, Royal blue.

* designates statistical significance to the reference dye, Royal Blue

**Fig 1:**
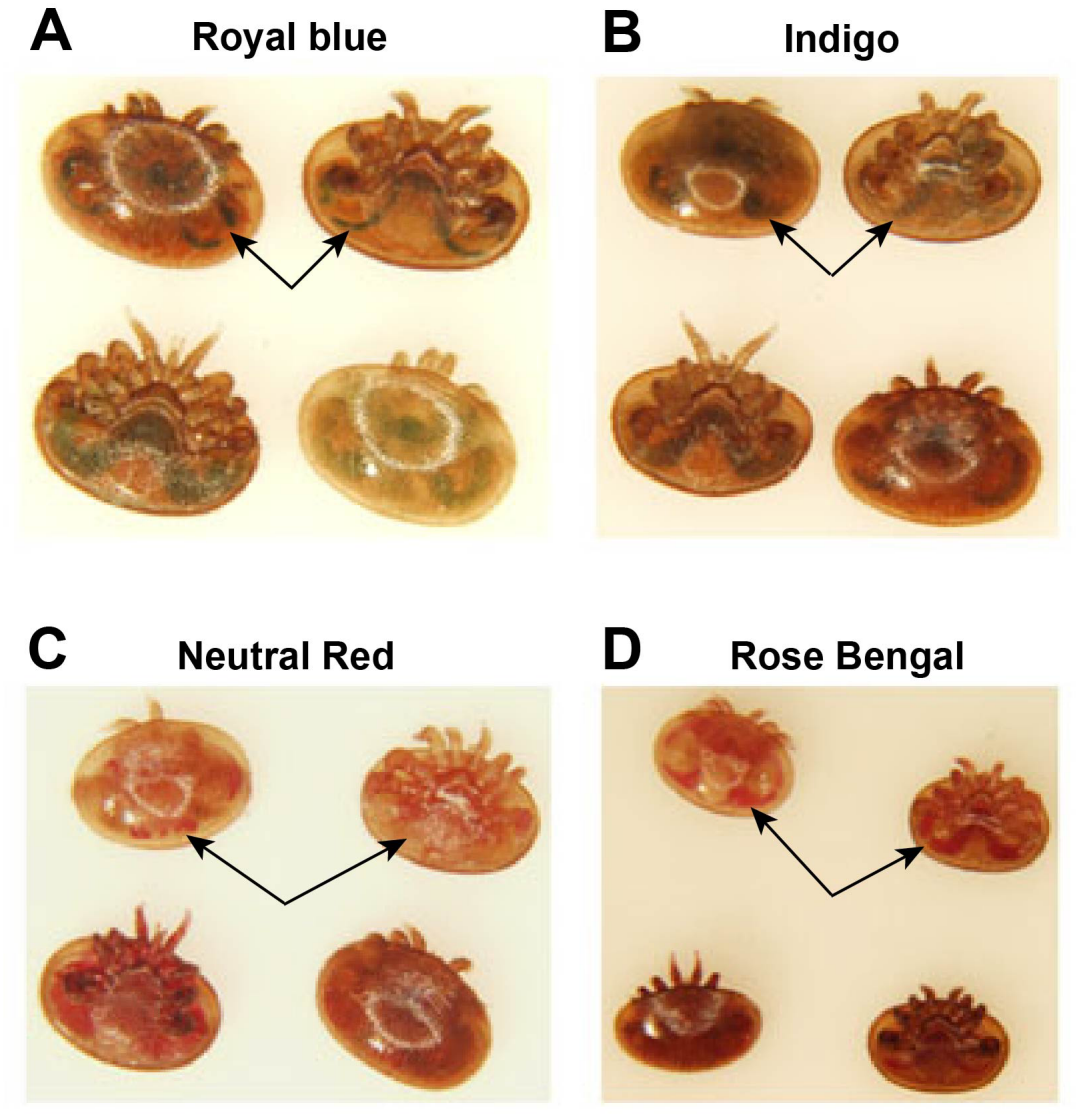
The visual detection of dyes in the Varroa mite after feeding. Of the four dyes that were ingested, (A) the Royal blue was the easiest to visually detect in the mite alimentary tract. The (B) Indigo, (C) Neutral Red and (D) Red Bengal were more difficult to differentiate from normal colors of the mites. Arrows point to regions within the mites where ingested dyes could be observed.

Various dyes were evaluated to determine the most effective method of visually identifying mites that had fed during the protocol including Royal Blue icing color, Day Glow, Indigo, Neutral Red and Rose Bengal. None of the mites exposed to the hemolymph treated with pink DayGlow showed evidence of feeding, ingestion of Neutral red resulted in 1.4% survival and Indigo only had 38.3% survive and feed (Table 3). The only dye that was not significantly different from Royal blue was Rose Bengal. However, the contrast in the color within the guts and other tissues was better with the Royal Blue than with Rose Bengal (Fig 1) and therefore Royal blue was used as the dye of choice as a convenient marker for visually scoring feeding in the protocol.

**Fig 2:**
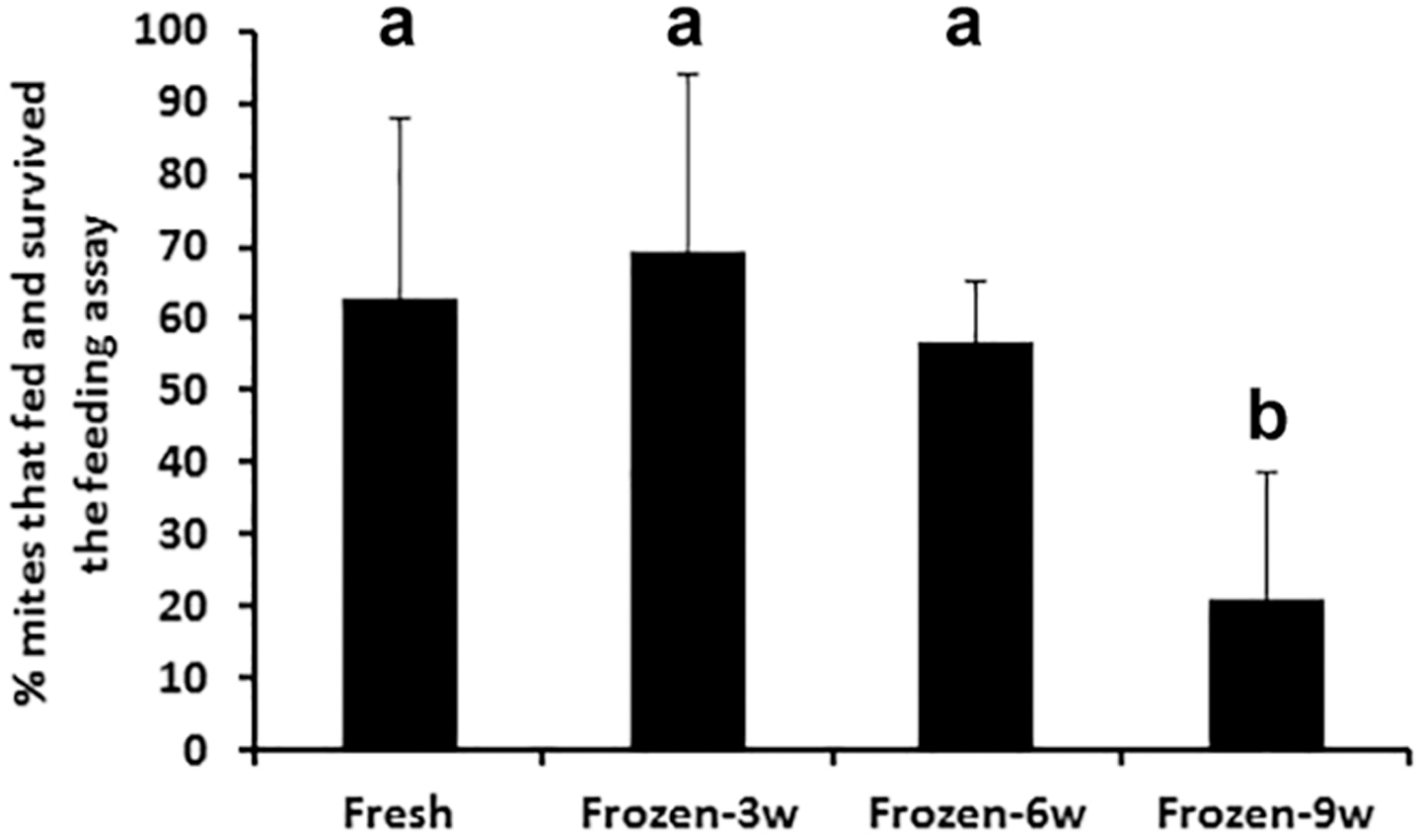
The effect of frozen storage on the efficacy of drone larva hemolymph to support feeding and survival of phoretic Varroa mites. Percentage of mites that fed and survived with hemolymph stored up to 6 weeks at -20°C was comparable to fresh hemolymph (F = 4.53, p = 0.0188, n = 4–6). Letter b designates statistical difference from a. Abbreviations: 3w = three weeks; 6w = six weeks; 9w = nine weeks.

To determine if the drone larva hemolymph could be preserved for use at a subsequent time, the effect of storage at -20°C was assessed. Drone larva hemolymph was prepared and treated as described above and frozen and kept at -20°C for 3 or 9 weeks. Fresh drone larva hemolymph (<16 h kept at 4°C) was prepared and then compared with 3, 6 or 9 week thawed drone larva hemolymph in a feeding protocol. Drone larva hemolymph frozen for up to 6 weeks had no significant effect on feeding or survival (Fig 2). However, the hemolymph stored at -20°C for 9 weeks showed a significant decline in efficacy where only 20.3% of the mites fed/survived the protocol.

To extend the conditions that included hemolymph feeding, mites were starved for 8 or 24 h then exposed to drone larva hemolymph for 8 or 24 h to assess survival. No significant differences were observed among various combinations of feeding protocols (>0.05). Based on these findings, the 24 h starvation and 8 h feeding on hemolymph with an overall survival of 46 ± 19.2% (n = 29) was selected as the preferred protocol conditions because it provided the highest survival with the longest feeding period.

To further stimulate feeding of the phoretic mites, cultured insect cells were added as a supplement to the feeding medium because free cells are present in hemolymph. The feeding medium was mixed 2:1 to contain 9 x 10^4^ cells per ml of either *B*. *mori* Bm5 or *D*. *melanogaster* S2 cells. To determine the amount of medium that the phoretic mites consumed during the feeding period, 1 x 10^10^ copies of pGem-Vdactin plasmidic DNA per μl were added to the treated hemolymph. There was a significant difference in the average consumption of hemolymph per mite among the various media supplements (F = 32.72, p<0.0001, n = 5). Phoretic mites fed on worker larva hemolymph alone consumed approximately 0.47 (SE = ± 0.06) nl during the 8 h feeding period with a 52.3 ± 15.0% (n = 9) fed/survival frequency. Although the phoretic mites that were fed with the hemolymph mixed with *B*. *mori* Bm5 cells consumed 1.10 (SE = ± 0.14) nl in 8 h, which was significantly higher than the hemolymph only group, the fed/survival frequency was 42.2 ± 17.6% (n = 10). In contrast, mites that fed on hemolymph supplemented with *D*. *melanogaster* S2 cells consumed significantly less, 0.12 (SE = ± 0.03) nl in 8 h, but also had significantly lower fed/survival frequencies (29.4 ± 17.4%) than the mites that fed on hemolymph only.

### Validation of the Varroa mite feeding protocol

The standard feeding protocol based on the previous assessments consisted of starving phoretic mites for 24 h in a container with a moist paper towel prior to placing 10–20 mites within an inverted 2 ml tube containing a small piece of cotton (2 ± 0.5 mg) moistened with 10–15 μl of treated larva hemolymph in the cap. The hemolymph diluted 2:1 with SIMC contained 10 or 15 mM ascorbic acid final concentration depending on the origin from drone or worker larvae, 2 μg/ml ampicillin, 2 μl Royal blue icing color per 100 μl of hemolymph and the desired experimental biological treatment at appropriate concentrations. The tubes were placed inside a Billups/Rothberg modular incubator with a moist paper towel in the bottom in an incubator at 28 ± 1°C and 65% relative humidity. After an 8 h feeding period, the mites were either observed to determine feeding and survival, and then stored at -80°C or processed immediately.

**Fig 3:**
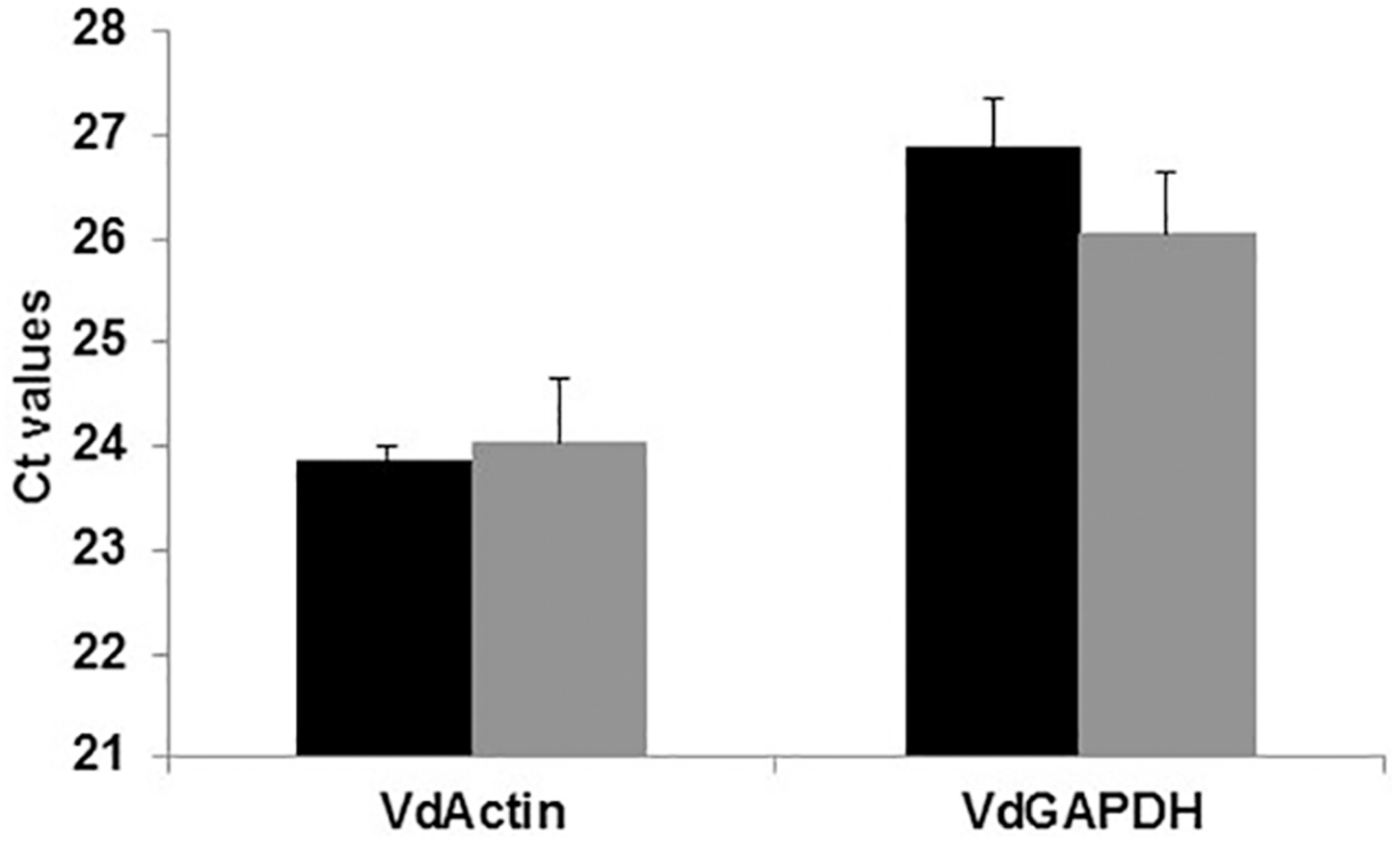
Comparison of transcript levels for the constitutively expressed actin and GAPDH genes in phoretic mites and those surviving the feeding protocol. Black bars are phoretic mites and gray bars represent mites completing feeding protocol. T bars represent standard error of the mean. Abbreviations: VdActin = actin and VdGAPDH = GAPDH.

To assess the impact of the feeding protocol on the physiology of the Varroa mites, transcript levels of two constitutively expressed genes, actin and GAPDH, were determined and compared between 0 h phoretic mites that were extracted immediately after collection and those that had been subjected to the standard feeding protocol. No significant differences in transcript levels of actin (t = 0.57, p = 0.5974, n = 3) or GAPDH (t = -1.89, p = 0.1334, n = 3) were observed between the untreated control mites and the mites that survived the feeding protocol (Fig 3).

**Fig 4:**
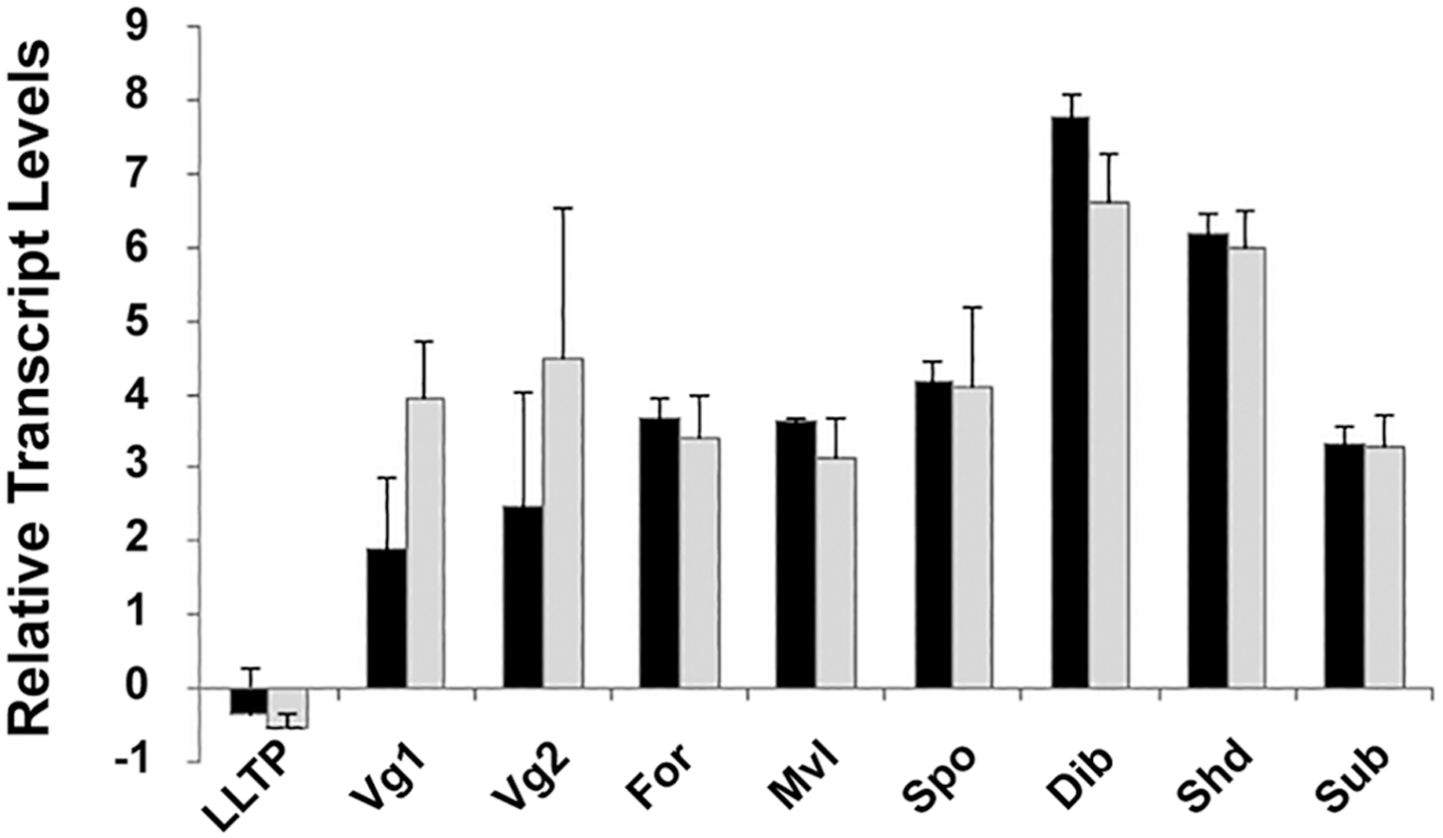
Relative transcript levels of nine selected genes in phoretic mites and in mites that had completed the feeding protocol. All transcript levels were normalized to the geometric mean of actin and GAPDH transcript levels and presented as fold differences as reported previously [59]. Black bars are phoretic mites and gray bars represent mites completing feeding protocol. T bars represent standard error of the mean. Abbreviations: LLTP = large lipid transport protein, Vg1 = vitellogenin 1, Vg2 = vitellogenin 2, For = foraging, Mvl = malvolio, Spo = spook, Dib = disembodied, Shd = shade and Sub = subolesin.

Besides the housekeeping genes, the transcript levels of 9 other Varroa mite genes responsive during the reproductive cycle were assessed. These genes included two vitellogenins (*Vd*Vg1, *Vd*Vg2) [33], a large lipid transfer protein (*Vd*LLTP) [34], foraging (*Vd*For) and malvolio (*Vd*Mvl) [60], spook (*Vd*spo), disembodied (*Vd*dib), shade (*Vd*shd) [59] and a co-transcription factor subolesin (*Vd*sub) (Cabrera and Shirk, unpublished data). Comparison of the transcript levels for each of the nine selected genes between untreated control phoretic mites and the phoretic mites that survived the feeding protocol showed that that there were no significant differences after completing the feeding protocol (VdLLTP: t = -1.13, p = 0.3228, n = 3; VdVg1: t = 1.97, p = 0.1195, n = 3; VdVg2: t = 1.12, p = 0.3260, n = 3; VdFor: t = -1.20, p = 0.2964, n = 3; VdMvl: t = -1.87, p = 0.1341, n = 3; Vdspo: t = -1.35, p = 0.2475, n = 3; Vddib: t = -1.70, p = 0.1640, n = 3; Vdshd: t = -1.12, p = 0.3274, n = 3; Vdshd: t = -1.12, p = 0.3247; Vdsub: t = -0.84, p = 0.4468, n = 3); Fig 4).

### Utilization of the feeding protocol to deliver biologically active materials

**Fig 5:**
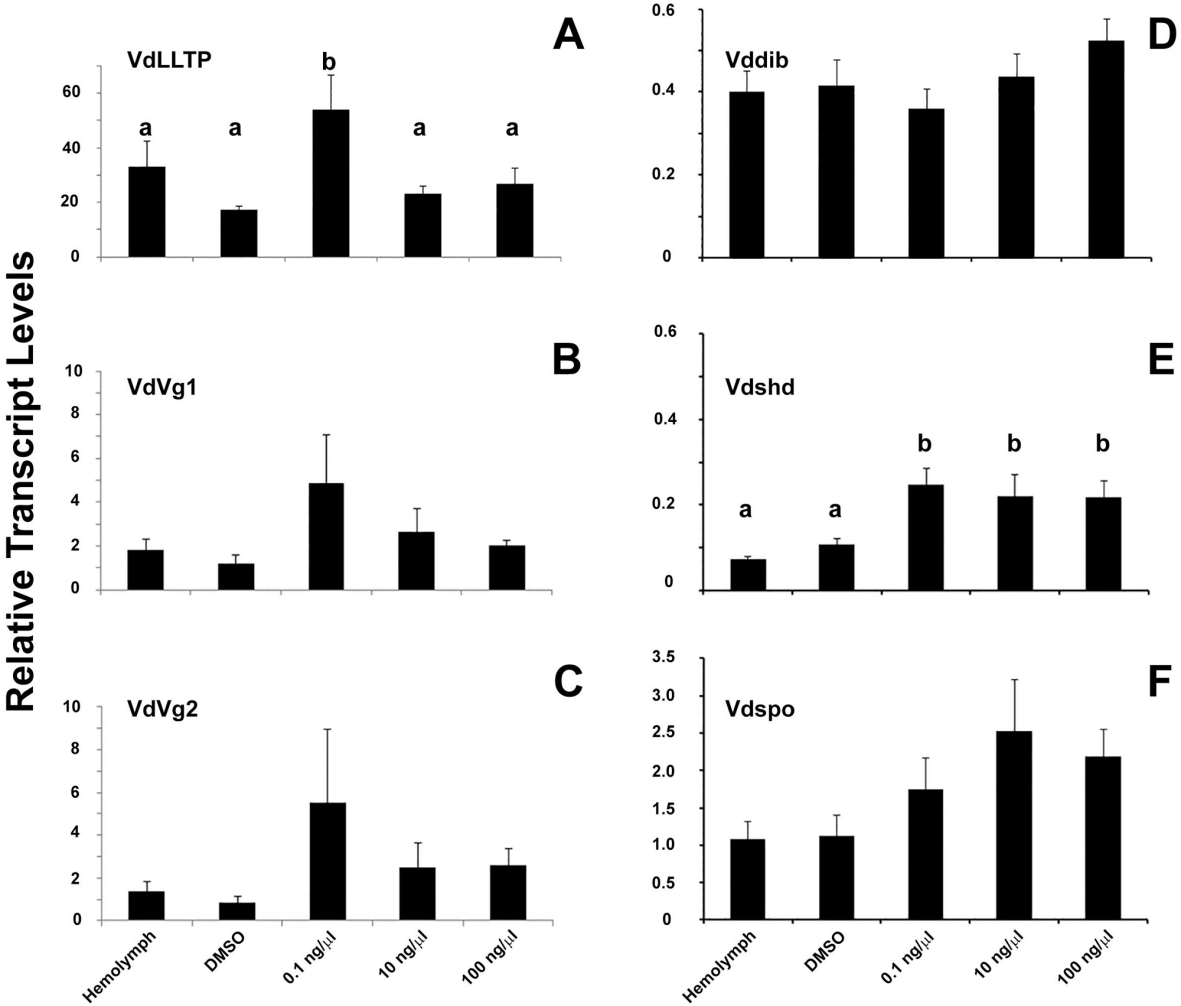
Elevation of selected transcripts by feeding on Tebufenozide. Phoretic Varroa mites were fed on media containing varying concentrations of Tebufenozide in the feeding protocol. RT-qPCR was conducted on the total RNA extracts from the surviving mites. Transcript levels were determined for the (A) large lipid transport protein (LLTP), (B) vitellogenin 1 (Vg1), (C) vitellogenin 2 (Vg2), (D) disembodied (Dib), (E) shade (Shd) and (F) spook (Spo) genes and normalized with the geometric mean for actin and GAPDH as reported previously [59]. T bars represent standard error of the mean. Letter “b” in panel A and E designate significant statistical difference from treatments with “a”. No statistical designations were added to the other panels because there were no statistical differences between treatments.

In order to determine if biologically active materials could be dispensed to the Varroa mites via the feeding protocol, Tebufenozide [62], an ecdysone analogue, was included in feeding media for the phoretic mites. Tebufenozide dissolved in DMSO at concentrations of 0.1, 10 and 1000 ng/μl dissolved in DMSO were included in the feeding medium. Even though the Tebufenozide was included in the feeding medium, it does not preclude topical absorption by contact on the cotton ball. At the end of the feeding protocol, total RNA was extracted and the transcript levels for *Vd*dib, *Vd*LLTP, *Vd*shd, *Vd*spo, *Vd*Vg1 and *Vd*Vg2 were determined and compared with base levels of transcripts from untreated mites that survived the feeding protocol. Inclusion of DMSO only in the feeding medium served as an experimental control. Transcript levels of *Vd*shd were significantly elevated when compared with the controls at all 3 tested Tebufenozide concentrations (F = 7.70 p = 0.004, n = 6, Dunnett’s critical value = 2.61744; Fig 5). Neither of the other two Halloween genes, *Vd*spo (F = 2.53, p = 0.0658, n = 6) or *Vd*dib (F = 1.19 p = 0.3399, n = 6), showed any significant change in transcript levels at any concentration of Tebufenozide from the control groups (Fig 5D and 5F). On the other hand, the transcript levels for *Vd*LLTP were significantly elevated at 0.1 ng/μl Tebufenozide, compared to the untreated control, but not at higher concentrations (F = 3.38, p = 0.02, n = 6, Dunnett’s critical value = 2.60688; Fig 5A). However, *Vd*Vg1 and *Vd*Vg2 transcript levels were not significantly different from the untreated control (Vg1: F = 0.51, p = 0.7310, n = 6), Vg2: F = 0.63, p = 0.6439, n = 6; Fig 5A and 5B).

## Discussion

The approaches to off-bee feeding for Varroa mites have typically been conducted by presenting media through a membrane [51–53]. In attempting to achieve production of a complete life cycle without feeding on bees, an artificial diet and feeding method was developed by placing the mites within a container that presented hemolymph or artificial diets in a stretched Parafilm pouch [51]. With this feeding platform, the mites of all developmental stages fed and the adult females laid eggs. However, the resulting progeny could not complete development. The introduction of a chitosan membrane covering the feeding medium resulted in a higher rate of feeding but only supported rearing for up to 5 days and was without initiation of oogenesis [53].

The feeding protocol developed here demonstrates that Varroa mites can feed and survive while off the bees without imposing the constraints of an intervening membrane as a component of the feeding platform. While, the intent of the feeding protocol was to provide a means of treating the Varroa mites with biologically active materials and not an attempt to establish a complete life cycle, this short-term protocol serves as a starting point to further develop a long-term off-host rearing protocol for Varroa mites. Utilizing cotton fiber as a substrate the mites were able to feed from the adhering liquid without being entrapped in or coated with the medium. The protocol led to feeding on the optimized feeding medium that included 5^th^ instar larval drone hemolymph and resulted in the fed/survival rates of 60% (Table 2 and Fig 2). These survival rates compare with those of those reported using the chitosan membrane chambers over similar time periods (see Fig 2 & Table 3 in [53]).

During the process of establishing the feeding protocol, various nutritional and behavioral parameters were assessed to maximize the short term feeding and survival rates. The initial attempts to establish feeding by Varroa mites were based on providing either a hemolymph solution or an artificial medium [51, 52]. Although the mites fed and incorporated materials from the artificial media into the eggs, the mites were not supported past 48 hours [51]. With the introduction of the chitosan membrane, the feeding medium provided to the mites was based on an artificial medium derived for *in vitro* growth of cultured honey bee cells [63] with or without the inclusion of larval bee hemolymph [53]. The chitosan membrane feeding assay did show that inclusion of hive materials, crude bees wax and 5^th^ instar bee larva hemolymph, produced the highest rate of feeding. The implications of the findings were that kairomones emanating from the honey bees wax and/or hemolymph were responsible for stimulating the feeding by the Varroa mites. The maximum feeding activities reported within this current study were associated with the inclusion of hemolymph as well. Basing the feeding medium on defined insect cell culture media showed that augmenting the medium with Schneider’s insect medium was as supportive of feeding and survival as media that included just last larval instar hemolymph. Additional supplementation of feeding medium with cultured insect cells showed that inclusion of the *B*. *mori* Bm5 cells led to a significant increase in feeding but did not result in greater survival. The Varroa mites may have a preference for the nature of cells they are feeding on because they fed and survived very poorly when supplemented with *D*. *melanogaster* S2 cells. Therefore, inclusion of cultured honeybee cells [64] may provide a more attractive and complete feeding medium. Because the cultured insect cells can be genetically transformed [65, 66], supplementation of the feeding medium can provide an additional means of introducing various constructs that will produce materials that can be tested for control of the mites.

Completion of the feeding protocol did not result in apparent physiological stress for the Varroa mites. Comparison of transcript levels of nine different genes between phoretic mites and those that had survived the protocol did not show any significant changes over the duration of the feeding. This lends confidence that the mites that feed are receiving adequate nutrition to sustain them and provide an opportunity to treat and measure the impact of biologically active materials on the mites. Addition of Tebufenozide [62, 67], a nonsteroidal ecdysteroid mimic, to the feeding medium resulted in a significant increase in the transcript levels for *shade*, a regulated terminal enzyme in the biosynthesis of 20-hydroxyecdysone [68], present in the Varroa mites [59]. The Tebufenozide also stimulated a significant increase in the large lipid transport transcript at the 0.1 ng level while at higher levels the transcript levels were lowered. This result may indicate that the ecdysteroid had a suppressive effect at higher levels similar to those observed during vitellogenesis in the Indian meal moth [69] and impact ecdysteroid levels in ovaries of the Mediterranean moth [70].

The availability of a protocol to deliver biologically active materials to the Varroa mites to identify targets for control and efficacy of alternative control measures is critical to establishing a control program for the Varroa mites [47]. The current RNAi protocol utilizes soaking the mites in a solution containing dsRNAs for delivery [48–50] thus providing a means of delivery for laboratory experimentation but of limited potential for field applications. The feeding protocol described in this report establishes a means to deliver and assess similar systems and molecules where oral administration could contribute as an additional avenue of application.

## Supporting information

S1 Fig A representative Varroa mite feeding assay test. For demonstration, ten mites (marked with arrows) were placed in ventilated 2 ml microtubes with a cotton ball treated with Royal blue, pink DayGlo or Neutral Red dye (tubes associated with dye balls identified with bars).
(TIF)

S1 Table Assessment of feeding substrate on percent survival of phoretic Varroa mites. (DOCX)

S2 Table Data for Varroa mite feeding assay. Collective data associated with treatments administered to Varroa mite specimens plus analyses performed here.
(XLSX)
