# Supplementary figures and images for: A feeding protocol for delivery of agents to assess development in Varroa mites
Source: PLoS One. 2017 Apr 27;12(4):e0176097. doi: 10.1371/journal.pone.0176097 (PMC5407785; doi:10.1371/journal.pone.0176097)

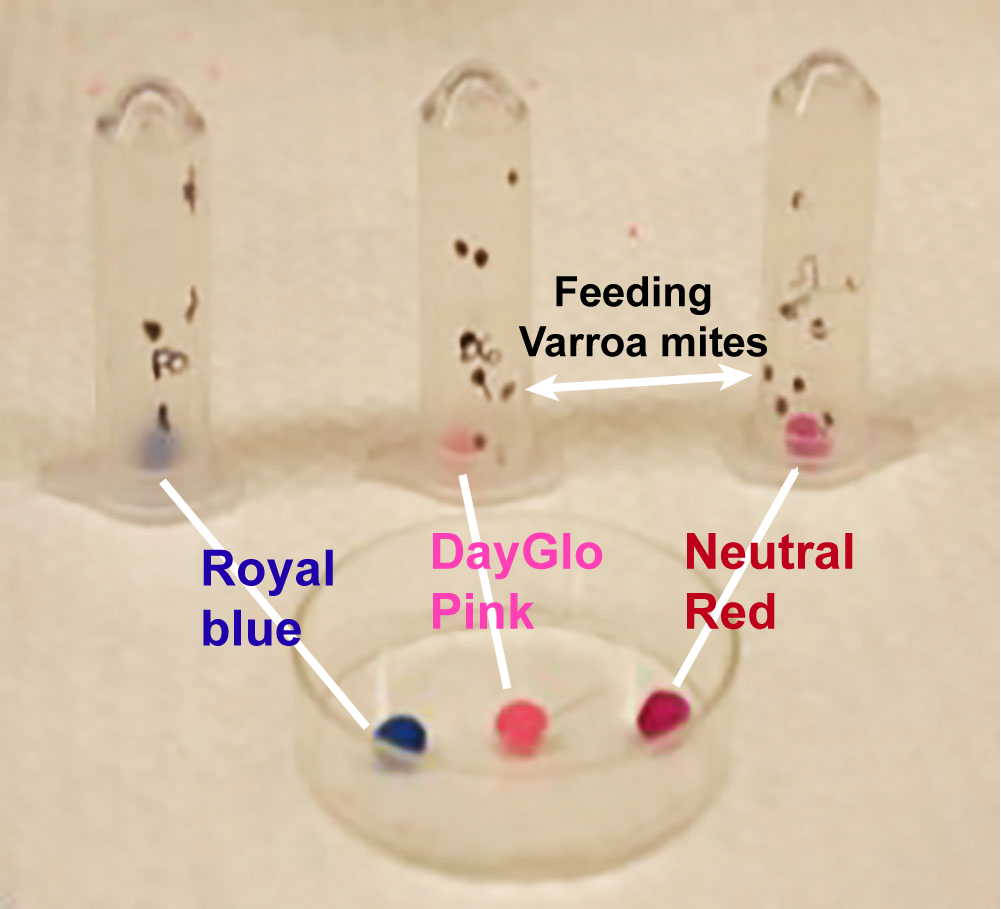

Supplement: S1 Fig — For demonstration, ten mites (marked with arrows) were placed in ventilated 2 ml microtubes with a cotton ball treated with Royal blue, pink DayGlo or Neutral Red dye (tubes associated with dye balls identified with bars). (TIF) [file pone.0176097.s001.tif]
