# Supplementary material for: A feeding protocol for delivery of agents to assess development in Varroa mites
Source: PLoS One. 2017 Apr 27;12(4):e0176097. doi: 10.1371/journal.pone.0176097 (PMC5407785; doi:10.1371/journal.pone.0176097)
Supplement: S1 Table — (DOCX) [file pone.0176097.s002.docx]

Supplemental Table 1. Assessment of feeding substrate on percent survival of phoretic Varroa mites.

| Feeding Substrate | n | Survived (24 h)  (%) |
| --- | --- | --- |
| Membrane | 40 | 0 |
| Parafilm | 30 | 50 |
| Nylon mesh | 20 | 25 |
| Cotton ball | 20 | 65 |
